# Smoothing approximation to the lower order exact penalty function for inequality constrained optimization

**DOI:** 10.1186/s13660-018-1723-x

**Published:** 2018-06-11

**Authors:** Shujun Lian, Nana Niu

**Affiliations:** School of Management Science, Qufu Normal University, Rizhao, China

**Keywords:** 90C30, Lower order penalty function, Inequality constrained optimization, Exact penalty function, Smoothing method

## Abstract

For inequality constrained optimization problem, we first propose a new smoothing method to the lower order exact penalty function, and then show that an approximate global solution of the original problem can be obtained by solving a global solution of a smooth lower order exact penalty problem. We propose an algorithm based on the smoothed lower order exact penalty function. The global convergence of the algorithm is proved under some mild conditions. Some numerical experiments show the efficiency of the proposed method.

## Introduction

Consider the following inequality constrained optimization problem: 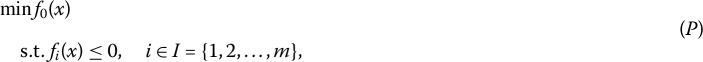 where $f_{i}:R^{n}\rightarrow R$, $i=0,1,\ldots,m$, are twice continuously differentiable functions. Throughout this paper, we use $X_{0}=\{x\in R^{n}| f_{i}(x)\leq0, i\in I\}$ to denote the feasible solution set.

This problem is widely applied in transportation, economics, mathematical programming, regional science, etc. [1–3], and it has received extensive attention on a related problem, for example, variational inequalities, equilibrium problem, minimizers of convex functions, etc. (see, e.g., [4–15]).

To solve problem (*P*), the penalty function methods have been introduced in many literature works (see, e.g., [16–24]). Zangwill [16] introduced the classical $l_{1}$ exact penalty function

1.1 $$ F_{1}(x,q)=f_{0}(x)+q\sum_{i=1}^{m} \max\bigl\{ f_{i}(x),0\bigr\} , $$

where $q>0$ is a penalty parameter, but it is not a smooth function. The corresponding penalty optimization problem is as follows: 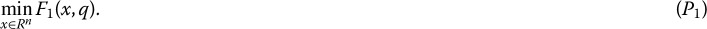 The non-smoothness of the function restricts the application of a gradient-type or Newton-type algorithm to solving problem ($P_{1}$). In order to avoid this shortcoming, the smoothing of the $l_{1}$ exact penalty function is proposed in [17, 18].

In addition, to overcome the non-smoothness of the function, the following smooth penalty function is proposed:

1.2 $$ F_{2}(x,q)=f_{0}(x)+q\sum_{i=1}^{m} \max\bigl\{ f_{i}(x),0\bigr\} ^{2}. $$

However, the function is non-exact.

Recently, Wu *et al.* [20] proposed the following low order penalty function:

1.3 $$ \varphi_{q,k}(x)=f_{0}(x)+q\sum_{i=1}^{m} \bigl(\max\bigl\{ f_{i}(x),0\bigr\} \bigr)^{k},\quad k \in(0,1), $$

and proved that the low order penalty function is exact under mild conditions. But this penalty function is non-smooth, too. When $k=1$, $\varphi _{q,k}(x)$ can be seen as the classical $l_{1}$ exact penalty function. The least exact penalty parameter corresponding to $k\in(0,1)$ is much less than that of the $l_{1}$ exact penalty function. This can avoid the defects of too large parameter *ρ* in the algorithm. Only for $k=\frac{1}{2}$, the smoothing of the lower order penalty function (1.3) is studied in [20] and [21]. In [24], a smoothing method of the low order penalty function (1.3) is given. We hope to study a new smoothing method for the low order penalty function (1.3) and compare it with the existing methods. With a different segmentation method, we will give a new piecewise smooth function and propose a new method to smooth the lower order penalty function (1.3) with $k\in[\frac{1}{2},1)$ in this paper.

The remainder of this paper is organized as follows. In Sect. 2, a new smoothing function is proposed. The error estimates are obtained among the optimal objective function values of the smoothed penalty problem, the non-smooth penalty problem, and the original problem. In Sect. 3, the corresponding algorithm is proposed to obtain an approximate solution to (*P*). The global convergence of the algorithm is proved. In Sect. 4, some numerical experiments are given to illustrate the efficiency of the algorithm. In Sect. 5, some conclusions are presented.

## A smoothing penalty function

For the lower order penalty problem 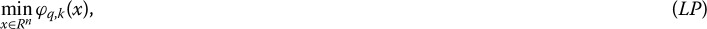 in order to establish the global exact penalization, the following assumption is given in [20]. We will consider the smoothing method under the following assumption.

### Assumption 2.1

1. $f_{0}(x)$ satisfies the coercive condition
  $$\lim_{\|x\|\rightarrow+\infty}f_{0}(x)=+\infty. $$
2. The optimal solution set $G(\mbox{($P$)})$ is a finite set.

Under Assumption 2.1, problem (*P*) is equivalent to the following problem: 
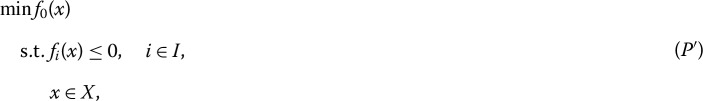
 where *X* is a box with $\operatorname{int}(X)\neq\emptyset$.

For any $k\in(0,1)$, penalty problem (*LP*) is equivalent to the following penalty problem: 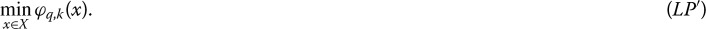


Now we consider a new smoothing technique to the lower order penalty function (1.3).

Let $p_{k}(t)=(\max\{t,0\})^{k}$, then

2.1 $$ \varphi_{q,k}(x)=f_{0}(x)+q\sum_{i=1}^{m}p_{k} \bigl(f_{i}(x)\bigr). $$

Define a function $p_{k,\epsilon}(t)$ ($\epsilon> 0$) by

2.2 $$ p_{k,\epsilon}(t)=\left \{ \textstyle\begin{array}{l@{\quad}l} 0, & \text{if } t\leq-\epsilon^{k},\\ \frac{k}{2}\epsilon^{-1}(t+\epsilon^{k})^{2}, & \text{if } {-}\epsilon^{k}< t< 0,\\ ({t+\epsilon})^{k}+\frac{k}{2}\epsilon^{2k-1}-\epsilon^{k},&\text{if } t\geq0, \end{array}\displaystyle \right . $$

where $\frac{1}{2}\leq k<1$. It is easy to see that $p_{k,\epsilon }(t)$ is continuously differentiable and

$$\lim_{\epsilon\rightarrow0^{+}}p_{k,\epsilon}(t)= p_{k}(t). $$

The following figure shows the process of function $p_{k,\epsilon}(t)$ approaching function $p_{k}(t)$.

**Figure 1:**
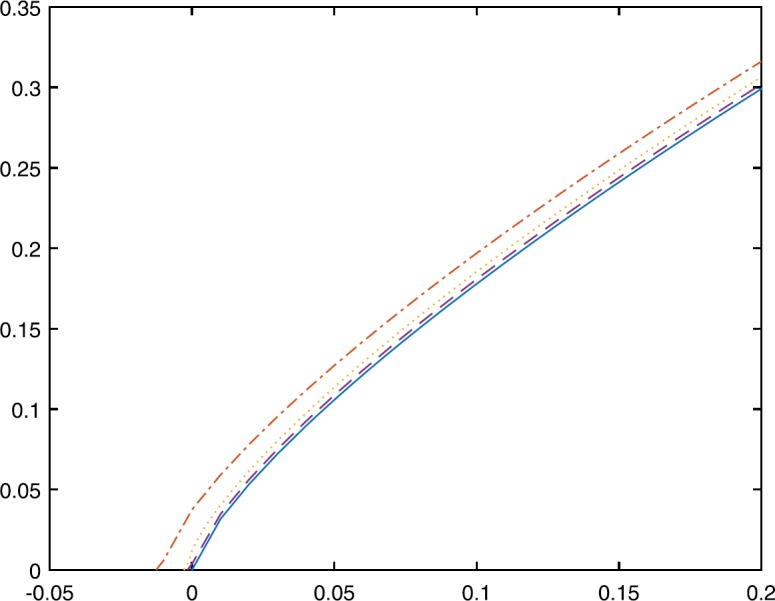
The behavior of $p_{k,\epsilon}(t)$ and $p_{k}(t)$

Figure 1 shows the behavior of $p_{\frac{3}{4},0.01}(t)$ (represented by the dash and dot line), $p_{\frac{3}{4},0.001}(t)$ (represented by the dot line), $p_{\frac{3}{4},0.0001}(t)$ (represented by the dash line), and $p_{\frac{3}{4}}(t)$ (represented by the solid line).

Based on this, we consider the following continuously differentiable penalty function:

2.3 $$ \varphi_{q,k,\epsilon}(x)=f_{0}(x)+q\sum_{i=1}^{m}p_{k,\epsilon} \bigl(f_{i}(x)\bigr), $$

where $\lim_{\varepsilon\rightarrow0^{+}} \varphi_{q,k,\epsilon }(x)=\varphi_{q,k}(x)$.

The corresponding optimization problem to $\varphi_{q,k,\epsilon}(x)$ is as follows: 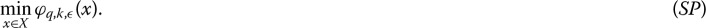


For problems (*P*), ($\mathit {LP}^{\prime}$), and (*SP*), we have the following conclusion.

### Lemma 2.1

*For any* $x\in X$, $\epsilon>0$, *and* $q >0$, *it holds that*

$$-\frac{k}{2}\epsilon^{2k-1}mq\leq\varphi_{q,k}(x)-\varphi _{q,k,\epsilon}(x)< \epsilon^{k}mq,\quad k\in\biggl[\frac{1}{2},1\biggr). $$

### Proof

For all $i\in I$, it holds that

$$p_{k}\bigl(f_{i}(x)\bigr)-p_{k,\epsilon} \bigl(f_{i}(x)\bigr)=\left \{ \textstyle\begin{array}{l@{\quad}l} 0, &\text{if } f_{i}(x)\leq-\epsilon^{k},\\ -\frac{k}{2}\epsilon^{-1}(f_{i}(x)+\epsilon^{k})^{2}, & \text{if } {-}\epsilon^{k} < f_{i}(x)< 0,\\ {f_{i}(x)^{k}-(f_{i}(x)+\epsilon})^{k}-\frac{k}{2}\epsilon ^{2k-1}+\epsilon^{k}, &\text{if } f_{i}(x)\geq0. \end{array}\displaystyle \right .$$

Set

$$F(t)=t^{k}-(t+\epsilon)^{k},\quad t\geq0. $$

Then

$$F^{\prime}(t)=k\bigl[t^{k-1}-(t+\epsilon)^{k-1}\bigr]. $$

It is easy to see that function $F(t)$ is monotonically increasing w.r.t. *t* due to that $k\in[\frac{1}{2},1)$. One has

$$-\epsilon^{k}\leq{f_{i}(x)^{k}- \bigl(f_{i}(x)+\epsilon}\bigr)^{k}\leq0,\quad \text{if } f_{i}(x)\geq0. $$

It follows that

$$-\frac{k}{2}\epsilon^{2k-1}\leq p_{k} \bigl(f_{i}(x)\bigr)-p_{k,\epsilon }\bigl(f_{i}(x)\bigr) \leq\epsilon^{k}, \quad \text{if }f_{i}(x)\geq0. $$

When $-\epsilon^{k}< f_{i}(x)<0$, one has

$$-\frac{k}{2}\epsilon^{2k-1}< p_{k}\bigl(f_{i}(x) \bigr)-p_{k,\epsilon}\bigl(f_{i}(x)\bigr)< 0. $$

So,

2.4 $$ -\frac{k}{2}\epsilon^{2k-1}\leq p_{k} \bigl(f_{i}(x)\bigr)-p_{k,\epsilon}\bigl(f_{i}(x)\bigr)< \epsilon^{k}, \quad\forall i\in I. $$

It follows from (2.1), (2.3), and (2.4) that

$$-\frac{k}{2}\epsilon^{2k-1}mq\leq\varphi_{q,k}(x)-\varphi _{q,k,\epsilon}(x)< \epsilon^{k}mq $$

by the fact that $q >0$. □

### Theorem 2.1

*For a positive sequence* $\{\varepsilon_{j}\}$, *which converges to* 0 *as* $j\to\infty$, *assume that* $x^{j}$ *is an optimal solution to* $\min_{x\in X} \varphi_{q,k,\epsilon_{j}}(x)$ *for some given* $q>0$, $k\in[\frac{1}{2},1)$. *If* *x̅* *is an accumulating point of sequence* $\{x^{j}\}$, *then* *x̄* *is an optimal solution to* $\min_{x\in X} \varphi_{q,k}(x)$.

### Proof

It follows from Lemma 2.1 that

2.5 $$ -\frac{k}{2}\epsilon_{j}^{2k-1}mq\leq \varphi_{q,k}(x)-\varphi _{q,k,\epsilon_{j}}(x)< \epsilon_{j}^{k}mq,\quad \forall x\in X. $$

Since $x_{j}$ is a solution to $\min_{x\in X} \varphi_{q,k,\epsilon_{j}}(x)$, one has

2.6 $$ \varphi_{q,k,\epsilon_{j}}(x_{j})\leq\varphi_{q,k,\epsilon_{j}}(x),\quad \forall x \in X. $$

It follows from (2.5) and (2.6) that

$$\begin{aligned}\varphi_{q,k}(x_{j})&< \varphi_{q,k,\epsilon_{j}}(x_{j})+ \epsilon_{j}^{k}mq \\ &\leq \varphi_{q,k,\epsilon_{j}}(x)+\epsilon_{j}^{k}mq \\ &\leq \varphi_{q,k}(x)+ \epsilon_{j}^{k}mq+ \frac{k}{2}\epsilon_{j}^{2k-1}mq. \end{aligned} $$

Letting $j\rightarrow\infty$ yields

$$\varphi_{q,k}(\bar{x})\leq\varphi_{q,k}(x). $$

Thus, *x̅* is an optimal solution to $\min_{x\in X} \varphi _{q,k}(x)$. □

### Theorem 2.2

*Let* $x_{q,k}^{*}\in X$ *be an optimal solution of problem* ($\mathit {LP}^{\prime}$), *and* $\bar{x}_{q,k,\epsilon}\in X$ *be an optimal solution of problem* (*SP*) *for some* $q>0$, $k\in[\frac{1}{2},1)$, *and* $\epsilon>0$. *Then*

$$-\frac{k}{2}\epsilon^{2k-1}mq\leq \varphi_{q,k} \bigl(x_{q,k}^{*}\bigr)-\varphi_{q,k,\epsilon}(\bar {x}_{q,k,\epsilon})< \epsilon^{k}mq. $$

### Proof

Under the hypothetical conditions, it holds that $\varphi_{q,k}(x_{q,k}^{*})\leq\varphi_{q,k}(x)$ and $\varphi _{q,k,\epsilon}(\bar{x}_{q,k,\epsilon})\leq\varphi_{q,k,\epsilon }(x)$, $\forall x\in X$.

Therefore, by Lemma 2.1, one has

$$-\frac{k}{2}\epsilon^{2k-1}mq\leq\varphi_{q,k} \bigl(x_{q,k}^{*}\bigr)-\varphi _{q,k,\epsilon}\bigl(x_{q,k}^{*} \bigr) \leq\varphi_{q,k}\bigl(x_{q,k}^{*}\bigr)- \varphi_{q,k,\epsilon}(\bar {x}_{q,k,\epsilon}) $$

and

$$\varphi_{q,k}\bigl(x_{q,k}^{*}\bigr)-\varphi_{q,k,\epsilon}(\bar {x}_{q,k,\epsilon}) \leq\varphi_{q,k}(\bar{x}_{q,k,\epsilon})- \varphi_{q,k,\epsilon }(\bar{x}_{q,k,\epsilon}) < \epsilon^{k}mq. $$

This completes the proof. □

### Corollary 2.1

*Suppose that Assumption* 2.1 *holds*, *and for any* $x\in G((P))$, *there exists* $\lambda^{*}\in R_{+}^{m}$ *such that the pair* $(x^{*},\lambda ^{*})$ *satisfies the second order sufficient condition* (*in* [20]). *Let* $x^{*}\in X $ *be an optimal solution of problem* (*P*) *and* $\bar{x}_{q,k,\epsilon}\in X $ *be an optimal solution of problem* (*SP*) *for some* $q>0$, $k\in[\frac{1}{2},1)$, *and* $\epsilon>0$. *Then there exists* $q^{*}>0$ *such that*, *for any* $q>q^{*}$,

$$-\frac{k}{2}\epsilon^{2k-1}mq\leq f_{0}\bigl(x^{*}\bigr)- \varphi_{q,k,\epsilon }(\bar{x}_{q,k,\epsilon})< \epsilon^{k}mq. $$

### Proof

It follows from Corollary 2.3 (in [20]) that $x^{*}\in X$ is an optimal solution of problem ($\mathit {LP}^{\prime}$). By Theorem 2.2, one has

$$-\frac{k}{2}\epsilon^{2k-1}mq\leq \varphi_{q,k}\bigl(x^{*} \bigr)-\varphi_{q,k,\epsilon}(\bar{x}_{q,k,\epsilon})< \epsilon^{k}mq. $$

Since $\sum_{i=1}^{m}p_{k}(f_{i}(x^{*}))=0$, it holds that

$$\varphi_{q,k}\bigl(x^{*}\bigr)=f_{0}\bigl(x^{*}\bigr)+q\sum _{i=1}^{m}p_{k} \bigl(f_{i}\bigl(x^{*}\bigr)\bigr)=f_{0}\bigl(x^{*}\bigr). $$

This completes the proof. □

### Definition 1

For $\epsilon>0$, if $x\in X $ is such that

$$f_{i}(x)\leq\epsilon, \quad i=1,2,\ldots,m, $$

then $x\in X $ is an *ϵ*-feasible solution of problem (*P*).

### Theorem 2.3

*Let* $x_{q,k}^{*}\in X $ *be an optimal solution of problem* ($\mathit {LP}^{\prime}$), *and* $\bar{x}_{q,k,\epsilon}\in X $ *be an optimal solution of problem* (*SP*) *for some* $q>0$, $k\in[\frac{1}{2},1)$, *and* $\epsilon>0$. *If* $x_{q,k}^{*}$ *is a* feasible *solution of problem* (*P*), *and* $\bar{x}_{q,k,\epsilon}$*is an* *ϵ*-*feasible solution of problem* (*P*), *then*

$$-\frac{k}{2}\epsilon^{2k-1}mq \leq f_{0} \bigl(x_{q,k}^{*}\bigr)-f_{0}(\bar {x}_{q,k,\epsilon})< \biggl(2^{k}\epsilon^{k}+\frac{k}{2} \epsilon^{2k-1}\biggr)mq. $$

### Proof

By (2.1), (2.3), and Theorem 2.2, one has

$$\begin{aligned}-\frac{k}{2}\epsilon^{2k-1}mq&\leq \varphi _{q,k}\bigl(x_{q,k}^{*}\bigr)-\varphi_{q,k,\epsilon}( \bar{x}_{q,k,\epsilon}) \\ &=f_{0}\bigl(x_{q,k}^{*}\bigr)+q\sum _{i=1}^{m}p_{k}\bigl(f_{i} \bigl(x_{q,k}^{*}\bigr)\bigr)-\Biggl(f_{0}(\bar {x}_{q,k,\epsilon})+q\sum_{i=1}^{m}p_{k,\epsilon} \bigl(f_{i}(\bar {x}_{q,k,\epsilon})\bigr)\Biggr) \\ &< \epsilon^{k}mq. \end{aligned} $$

Since $\sum_{i=1}^{m}p_{k}(f_{i}(x_{q,k}^{*}))=0$, it holds that

2.7 $$\begin{aligned}[b] -\frac{k}{2}\epsilon^{2k-1}mq+q\sum_{i=1}^{m}p_{k,\epsilon} \bigl(f_{i}(\bar{x}_{q,k,\epsilon})\bigr) &\leq f_{0}\bigl(x_{q,k}^{*}\bigr)-f_{0}( \bar{x}_{q,k,\epsilon}) \\ &< \epsilon^{k}mq+q\sum_{i=1}^{m}p_{k,\epsilon} \bigl(f_{i}(\bar {x}_{q,k,\epsilon})\bigr).\end{aligned} $$

Note that

$$f_{i}(\bar{x}_{q,k,\epsilon})\leq\epsilon,\quad i\in I. $$

Thus, it follows from (2.2) that

2.8 $$ 0\leq q\sum_{i=1}^{m}p_{k,\epsilon} \bigl(f_{i}(\bar {x}_{q,k,\epsilon})\bigr) \leq\biggl(2^{k} \epsilon^{k}+\frac{k}{2}\epsilon^{2k-1}-\epsilon ^{k}\biggr)mq. $$

By (2.7) and (2.8), one has

$$-\frac{k}{2}\epsilon^{2k-1}mq \leq f_{0} \bigl(x_{q,k}^{*}\bigr)-f_{0}(\bar {x}_{q,k,\epsilon})< \biggl(2^{k}\epsilon^{k}+\frac{k}{2} \epsilon^{2k-1}\biggr)mq . $$

 □

Theorems 2.1 and 2.2 show that an optimal solution of (*SP*) is also an approximate optimal solution of ($\mathit {LP}^{\prime}$) when the error *ϵ* is sufficiently small. By Theorem 2.3, an optimal solution of (*SP*) is an approximately optimal solution of (*P*) if the optimal solution of (*SP*) is an *ϵ*-feasible solution of (*P*).

## A smoothing method

Based on the discussion in the last section, we can design an algorithm to obtain an approximate optimal solution of (*P*) by solving (*SP*).

### Algorithm 3.1

Step 1. Take $x^{0}$, $\epsilon_{0}>0$, $0< a<1$, $q_{0}>0$, $b>1$, $\epsilon>0$, and $k\in[\frac{1}{2},1)$, let $j=0$ and go to Step 2.
Step 2. Solve $\min_{x\in R^{n}} \varphi_{q_{j},k,\epsilon_{j}}(x)$ starting at $x^{j}$. Let $x^{j+1}$ be the optimal solution ($x^{j+1}$ can be obtained by a quasi-Newton method).
Step 3. Let $\epsilon_{j+1}=a\epsilon_{j}$, $q_{j+1}=b q_{j}$, and $j=j+1$, then go to Step 2.

### Remark

Since $0< a<1$ and $b>1$, let $a^{2k-1}b<1$, as $j\rightarrow+\infty$, the sequence $\{\epsilon_{j}\}$ is gradually decreased to 0, the sequence $\{q_{j}\}$ is gradually increased to +∞ and $\{\epsilon_{j}^{2k-1}q_{j}\} $ is gradually decreased to 0.

Under some mild conditions, the following conclusion shows the global convergence of Algorithm 3.1.

### Theorem 3.1

*Suppose that Assumption* 2.1 *holds*, *and for any* $\epsilon\in(0,\epsilon_{0}]$, $q\in[q_{0},+\infty)$, *the solution set of* $\min_{x\in R^{n}} \varphi_{q,k,\epsilon}(x)$ *is nonempty*. *If* $\{x^{j+1}\}$ *is the sequence generated by Algorithm *3.1 *satisfying* $a^{2k-1}b<1$, *and the sequence* $\{\varphi_{q_{j},k,\epsilon_{j}}(x^{j+1})\}$ *is bounded*, *then*

1. $\{x^{j+1}\}$ *is bounded*.
2. *Any limit point of* $\{x^{j+1}\}$ *is an optimal solution of* (*P*).

### Proof

(1) It follows from (2.3) that

3.1 $$ \varphi_{q_{j},k,\epsilon _{j}}\bigl(x^{j+1}\bigr)=f_{0} \bigl(x^{j+1}\bigr)+q_{j}\sum_{i=1}^{m}p_{k,\epsilon _{j}} \bigl(f_{i}\bigl(x^{j+1}\bigr)\bigr),\quad j=0,1,2,\ldots. $$

By hypothesis, there exists some number L such that

3.2 $$ L>\varphi_{q_{j},k,\epsilon_{j}}\bigl(x^{j+1}\bigr),\quad j=0,1,2,\ldots. $$

For the sake of contradiction, suppose that $\{x^{j+1}\}$ is unbounded. Without loss of generality, we assume that $\|x^{j+1}\|\rightarrow\infty$ as $j\rightarrow \infty$.

By (2.2), (3.1), and (3.2), one has

$$L>f_{0}\bigl(x^{j+1}\bigr), \quad j=0,1,2,\ldots, $$

which results in a contradiction with Assumption 2.1(1).

(2) Without loss of generality, we assume $x^{j+1}\rightarrow x^{*}$ as $j\rightarrow\infty$.

To prove $x^{*}$ is the optimal solution of (*P*), it is only needed to show that $x^{*}\in X_{0}$ and $f_{0}(x^{*})\leq f_{0}(x)$, $\forall x\in X_{0}$.

To show that $x^{*}\in X_{0}$, we outline a proof by contradiction. We presuppose that $x^{*}\notin X_{0}$, then there exist $\delta_{0}>0$, $i_{0}\in I$, and the subset $J \subset N$ such that

$$f_{i_{0}}\bigl(x^{j+1}\bigr)\geq\delta_{0}> \epsilon_{j},\quad \forall j\in J, $$

where *N* is the natural number set.

By Step 2, (2.2), and (2.3), for any $x\in X_{0}$, one has

$$\begin{aligned}f_{0}\bigl(x^{j+1} \bigr)+q_{j}{\biggl((\delta_{0}+\epsilon _{j})^{k}+\frac{k}{2}\epsilon_{j}^{2k-1}- \epsilon_{j}^{k}\biggr)} &\leq\varphi_{q_{j},k,\epsilon_{j}} \bigl(x^{j+1}\bigr) \\ &\leq\varphi_{q_{j},k,\epsilon_{j}}(x) \\ &\leq f_{0}(x)+m\frac{k}{2}\epsilon_{j}^{2k-1}q_{j}. \end{aligned} $$

It follows that

$$f_{0}\bigl(x^{j+1}\bigr)+q_{j}{\bigl(( \delta_{0}+\epsilon_{j})^{k}-\epsilon _{j}^{k}\bigr)}\leq f_{0}(x)+(m-1) \frac{k}{2}\epsilon_{j}^{2k-1}q_{j},\quad \forall x \in X_{0}, $$

which contradicts with $q_{j}\rightarrow +\infty, \epsilon_{j}\rightarrow 0$, and $\epsilon_{j}^{2k-1}q_{j}\rightarrow 0$, as $j\rightarrow\infty$. Then we have that $x^{*}\in X_{0}$.

Next, we show that $f_{0}(x^{*})\leq f_{0}(x)$, $\forall x\in X_{0}$.

For this, by Step 2, (2.2), and (2.3), it holds that

$$f_{0}\bigl(x^{j+1}\bigr)\leq\varphi_{q_{j},k,\epsilon_{j}} \bigl(x^{j+1}\bigr) \leq\varphi_{q_{j},k,\epsilon_{j}}(x)\leq f_{0}(x)+m\frac {k}{2}\epsilon_{j}^{2k-1}q_{j},\quad \forall x\in X_{0}. $$

Letting $j\rightarrow\infty$ yields that

$$f_{0}\bigl(x^{*}\bigr)\leq f_{0}(x). $$

Therefore, any limit point of $\{x^{j+1}\}$ is an optimal solution of (*P*). □

## Numerical examples

In this section, we will do some numerical experiments to show the efficiency of Algorithm 3.1.

### Example 4.1

Consider the following optimization problem considered in [18, 22, 23]:

$$\begin{gathered}\min f_{0}(x)=x_{1}^{2}+x_{2}^{2}+2x_{3}^{2}+x_{4}^{2}-5x_{1}-5x_{2}-21x_{3}+7x_{4} \\ \quad\text{s.t. } f_{1}(x)=2x_{1}^{2}+x_{2}^{2}+x_{3}^{2}+2x_{1}+x_{2}+x_{4}-5 \leq0, \\ \quad\phantom{\text{s.t. }} f_{3}(x)=x_{1}^{2}+x_{2}^{2}+x_{3}^{2}+x_{4}^{2}+x_{1}-x_{2}+ x_{3}-x_{4}-8 \leq0, \\ \quad\phantom{\text{s.t. }} f_{3}(x)= x_{1}^{2}+2x_{2}^{2}+x_{3}^{2}+2x_{4}^{2}-x_{1}-x_{4}-10 \leq0. \end{gathered} $$

For this problem, we let $k=\frac{3}{4}$, $\epsilon_{0}=0.01$, $a=0.01$, $q_{0}=1$, $b =2$, $\epsilon=10^{-16}$. With different starting points, numerical results of Algorithm 3.1 are shown in Tables 1, 2, and 3.

**Table 1:** Numerical results for Example 4.1 with $x^{0}=(0,0,0,0)$

*j*	$x^{j+1}$	$q_{j}$	$\epsilon_{j}$	$f_{1}(x^{j+1})$	$f_{2}(x^{j+1})$	$f_{3}(x^{j+1})$	$f_{0}(x^{j+1})$
0	(0.185009,0.804369,2.015460,−0.952409)	1	0.01	−4.797079	−0.00109	−2.028111	−44.225926
1	(0.169902,0.835670,2.008151,−0.965196)	2	0.0001	−9.748052	−9.337847	−1.883271	−44.231252

**Table 2:** Numerical results for Example 4.1 with $x^{0}=(2,0,3.5,0)$

*j*	$x^{j+1}$	$q_{j}$	$\epsilon_{j}$	$f_{1}(x^{j+1})$	$f_{2}(x^{j+1})$	$f_{3}(x^{j+1})$	$f_{0}(x^{j+1})$
0	(0.169693,0.835634,2.008291,−0.965082)	1	0.01	−9.502428	−8.676884	−1.883244	−44.231403

**Table 3:** Numerical results for Example 4.1 with $x^{0}=(2,2,2,0.5)$

*j*	$x^{j+1}$	$q_{j}$	$\epsilon_{j}$	$f_{1}(x^{j+1})$	$f_{2}(x^{j+1})$	$f_{3}(x^{j+1})$	$f_{0}(x^{j+1})$
0	(0.169691,0.835633,2.008294,−0.965080)	1	0.01	−9.502279	−8.676796	−1.883249	−44.231403

From Tables 1, 2, 3, we know that the obtained approximate optimal solutions are similar, which shows that the numerical result of Algorithm 3.1 does not depend on the section of the starting points for this example. In [18], the objective function value $f_{0}(x^{*})=-44.23040$ was obtained in the forth iteration. From the numerical results given in [22], we know that the optimal solution is $x^{*}=(0.1585001,0.8339736,2.014753,-0.959688 )$ with the objective function value $f_{0}(x^{*})=-44.22965$. In [23], the objective function value obtained in the 25th iteration is $f_{0}(x^{*})=-44$. Hence, the numerical results obtained by Algorithm 3.1 are better than the numerical results given in [18, 22, 23] for this example.

### Example 4.2

Consider the following problem considered in [17]:

$$\begin{gathered}\min f_{0}(x)=-2x_{1}-6x_{2}+x_{1}^{2}-2x_{1}x_{2}+2x_{2}^{2} \\ \quad\text{s.t. }f_{1}(x)=x_{1}+x_{2}-2 \leq0, \\ \quad\phantom{\text{s.t. }} f_{2}(x)=-x_{1}+2x_{2}-2 \leq0, \\ \quad\phantom{\text{s.t. }} x_{1}, x_{2}\geq0. \end{gathered} $$

For this problem, we let $x^{0}=(0,0)$, $\epsilon_{0}=0.001$, $a=0.001$, $q_{0}=2$, $b =10$, $\epsilon=10^{-16}$. With different *k*, numerical results of Algorithm 3.1 are shown in Tables 4, 5, and 6.

**Table 4:** Numerical results for Example 4.2 with $k=\frac{3}{4}$

*j*	$x^{j+1}$	$q_{j}$	$\epsilon_{j}$	$f_{1}(x^{j+1})$	$f_{2}(x^{j+1})$	$f_{0}(x^{j+1})$
0	(3.4217,2.7082)	2	0.001	4.1300	−0.0053	−15.2492
1	(0.8022,1.1978)	20	0.000001	0.0000	−0.4066	−7.1999

**Table 5:** Numerical results for Example 4.2 with $k=\frac{3}{5}$

*j*	$x^{j+1}$	$q_{j}$	$\epsilon_{j}$	$f_{1}(x^{j+1})$	$f_{2}(x^{j+1})$	$f_{0}(x^{j+1})$
0	(4.0607,3.0227)	2	0.001	5.0834	−0.0153	−16.0434
1	(0.8027,1.1971)	20	0.000001	−0.0003	−0.4086	−7.1992

**Table 6:** Numerical results for Example 4.2 with $k=\frac{8}{9}$

*j*	$x^{j+1}$	$q_{j}$	$\epsilon_{j}$	$f_{1}(x^{j+1})$	$f_{2}(x^{j+1})$	$f_{0}(x^{j+1})$
0	(2.6356,2.3168)	2	0.001	2.9523	−0.0020	−13.7027
1	(0.8005,1.1995)	20	0.000001	0.0000	−0.4015	−7.2000

From Tables 4, 5, 6, we can see that almost the same approximate optimal solutions are obtained for different *k* in this example. The objective function value is similar to the objective function value $f_{0}(x^{*})=-7.2000$ with $x^{*}=(0.8000,1.2000)$ obtained in the forth iteration in [17].

### Example 4.3

Consider the following problem considered in [24] and [25] (Test Problem 6 in Sect. 4.6):

$$\begin{gathered}\min f_{0}(x)=-x_{1}-x_{2} \\ \quad\text{s.t. }f_{1}(x)=x_{2}-2x_{1}^{4}+8x_{1}^{3}-8x_{1}^{2}-2 \leq 0, \\ \quad\phantom{\text{s.t. }} f_{2}(x)=x_{2}-4x_{1}^{4}+32x_{1}^{3}-88x_{1}^{2}+96x_{1}-36 \leq0, \\ \quad\phantom{\text{s.t. }}0\leq x_{1} \leq3,\qquad 0\leq x_{2} \leq4. \end{gathered} $$

For this problem, we set $k=\frac{2}{3}$, $x^{0}=(0,0)$, $\epsilon_{0}=0.01$, $a=0.01$, $q_{0}=5$, $b =2$, $\epsilon=10^{-16}$. The numerical results of Algorithm 3.1 are shown in Table 7.

**Table 7:** Numerical results for Example 4.3 with $x^{0}=(0,0)$

*j*	$x^{j+1}$	$q_{j}$	$\epsilon_{j}$	$f_{1}(x^{j+1})$	$f_{2}(x^{j+1})$	$f_{0}(x^{j+1})$
0	(2.329795,3.133729)	5	10^−2^	−0.047009	−0.043471	−5.463524
1	(2.329238,3.173320)	10	10^−4^	−0.002868	−0.006501	−5.502557
2	(2.329452,3.177637)	20	10^−6^	−0.000302	−0.001176	−5.507089
3	(2.329626,3.177558)	40	10^−8^	−0.001802	−0.000436	−5.507185

We set $k=\frac{8}{9}$, $x^{0}=(1.0,1.5)$, $\epsilon_{0}=0.1$, $a=0.1$, $q_{0}=5$, $b =3$, $\epsilon=10^{-16}$. The numerical results of Algorithm 3.1 are shown in Table 8.

**Table 8:** Numerical results for Example 4.3 with $x^{0}=(1.0,1.5)$

*j*	$x^{j+1}$	$q_{j}$	$\epsilon_{j}$	$f_{1}(x^{j+1})$	$f_{2}(x^{j+1})$	$f_{0}(x^{j+1})$
0	(2.330261,3.061875)	5	10^−1^	−0.1226776	−0.1131323	−5.392137
1	(2.329664,3.161611)	15	10^−2^	−0.018055	−0.016207	−5.491275
2	(2.329639,3.171941)	45	10^−3^	−0.007524	−0.005993	−5.501580
3	(2.329560,3.177804)	135	10^−4^	−0.001013	−0.000503	−5.507363
4	(2.329593,3.177793)	405	10^−5^	−0.001297	−0.000357	−5.507386
5	(2.329622,3.177781)	1215	10^−6^	−0.001544	−0.000234	−5.507403

We set $k=\frac{3}{4}$, $x^{0}=(2,0.5)$, $\epsilon_{0}=0.00001$, $a=0.001$, $q_{0}=2$, $b =10$, $\epsilon=10^{-16}$. The numerical results of Algorithm 3.1 are shown in Table 9.

**Table 9:** Numerical results for Example 4.3 with $x^{0}=(2,0.5)$

*j*	$x^{j+1}$	$q_{j}$	$\epsilon_{j}$	$f_{1}(x^{j+1})$	$f_{2}(x^{j+1})$	$f_{0}(x^{j+1})$
0	(2.330460,3.179900)	2	10^−5^	−0.006287	0.005832	−5.510360
1	(2.329672,3.179735)	20	10^−8^	−0.000001	0.001957	−5.509408
2	(2.329672,3.179735)	200	10^−11^	−0.000000	0.001957	−5.509407
3	(2.329541,3.178391)	2000	10^−14^	−0.000000	−0.000000	−5.507933

In [24], with three different starting points, similar numerical results are given with ${k=\frac{2}{3}}$. The optimal solution $(2.329517, 3.178421)$ is given with the objective function value −5.507938. In [25], the optimal solution $(2.3295, 3.1783)$ is given with the objective function value −5.5079. The numerical results of Example 4.3 are similar to the numerical results of [24] and [25] in this example.

From Tables 7, 8, 9, we can see that we need to adjust the parameters $q_{0}$, $\epsilon_{0}$, *a*, *b* to get the better numerical results with different *k* and $x^{0}$. Usually, $\epsilon_{0}$ may be 0.5, 0.1, 0.01, 0.001, or smaller digits, and $a=0.5, 0.1,0.01$, or 0.001. $q_{0}$ may be 1, 2, 3, 5, 10, 100, or larger digits, and $b= 2, 3, 5, 10$, or 100.

## Concluding remarks

In this paper, we proposed a method to smooth the lower order exact penalty function with $k\in[\frac{1}{2},1)$ for inequality constrained optimization. Furthermore, we proved that the algorithm based on the smoothed penalty functions is globally convergent under mild conditions. The given numerical experiments show that the algorithm is effective.
